# Characterizing Families of Spectral Similarity Scores and Their Use Cases for Gas Chromatography–Mass Spectrometry Small Molecule Identification

**DOI:** 10.3390/metabo13101101

**Published:** 2023-10-21

**Authors:** David J. Degnan, Javier E. Flores, Eva R. Brayfindley, Vanessa L. Paurus, Bobbie-Jo M. Webb-Robertson, Chaevien S. Clendinen, Lisa M. Bramer

**Affiliations:** 1 Biological Sciences Division, Pacific Northwest National Laboratory, Richland, WA 99354, USA; david.degnan@pnnl.gov (D.J.D.); javier.flores@pnnl.gov (J.E.F.); bj@pnnl.gov (B.-J.M.W.-R.); 2 Artificial Intelligence and Data Analytics Division, Pacific Northwest National Laboratory, Richland, WA 99354, USA; eva.brayfindley@pnnl.gov; 3 Environmental and Molecular Sciences Division, Pacific Northwest National Laboratory, Richland, WA 99354, USA; vanessa.paurus@pnnl.gov (V.L.P.); chaevien.clendinen@pnnl.gov (C.S.C.)

**Keywords:** distance metrics, metabolomics, machine learning, One Health

## Abstract

Metabolomics provides a unique snapshot into the world of small molecules and the complex biological processes that govern the human, animal, plant, and environmental ecosystems encapsulated by the One Health modeling framework. However, this “molecular snapshot” is only as informative as the number of metabolites confidently identified within it. The spectral similarity (SS) score is traditionally used to identify compound(s) in mass spectrometry approaches to metabolomics, where spectra are matched to reference libraries of candidate spectra. Unfortunately, there is little consensus on which of the dozens of available SS metrics should be used. This lack of standard SS score creates analytic uncertainty and potentially leads to issues in reproducibility, especially as these data are integrated across other domains. In this work, we use metabolomic spectral similarity as a case study to showcase the challenges in consistency within just one piece of the One Health framework that must be addressed to enable data science approaches for One Health problems. Here, using a large cohort of datasets comprising both standard and complex datasets with expert-verified truth annotations, we evaluated the effectiveness of 66 similarity metrics to delineate between correct matches (true positives) and incorrect matches (true negatives). We additionally characterize the families of these metrics to make informed recommendations for their use. Our results indicate that specific families of metrics (the Inner Product, Correlative, and Intersection families of scores) tend to perform better than others, with no single similarity metric performing optimally for all queried spectra. This work and its findings provide an empirically-based resource for researchers to use in their selection of similarity metrics for GC-MS identification, increasing scientific reproducibility through taking steps towards standardizing identification workflows.

## 1. Introduction

One Health is an analytic framework that recognizes the complex dependencies that exist between the environment and its member organisms, with particular focus on the impact of these interrelated biosystems on health outcomes [1]. Adopting a One Health approach requires the collection of data at multiple scales, ranging from the macro (e.g., climate, environmental hazards) to the micro (e.g., individual-level omic biomarkers). These diverse forms of data each present unique challenges in processing and analysis, which must be addressed before one even considers integrating these data within a One Health model.

In the present work, we focus on metabolomics data, which (1) are crucial towards achieving a mechanistic understanding of the interactions between the biological entities thriving within global ecosystems, and (2) provide a useful case study in the challenges of dataset consistency within just one small component of larger One Health systems. Several authors have highlighted the use of ‘omics data in One Health approaches [2,3,4,5,6,7,8]. The authors in [8], for example, demonstrate the importance of metabolomics to One Health by providing several examples in human and veterinary health of the impacts made by metabolomics. These examples include the use of metabolomic profiling to identify nutritional biomarkers for cancer, diabetes, and other chronic or metabolic diseases; for identifying toxicological biomarkers in dogs; and for demonstrating differences in production of greenhouse gas precursors across personalized diets for grazing animals (e.g., cows). However, the value offered by metabolomics rests entirely on the ability to accurately identify metabolites within obtained samples in a consistent manner.

Gas Chromatography coupled to Mass Spectrometry (GC-MS) is a classic method for the high throughput, untargeted measurement of metabolites and other small molecules. Briefly, samples are vaporized and separated in a capillary column where each component is eluted, ionized, fragmented, and analyzed with a mass analyzer. The results are a series of mass spectra, which is the intensity of each fragment versus the mass to charge ratio (*m*/*z*) per elution (retention) time of each component. The compounds represented within each of these output mass spectra are identified by matching their spectra to a reference library of known candidate spectra, where the quality of each candidate match is ranked by some spectral similarity (SS) metric. As of this publication, there are many metrics which compare scaled measured intensities (called abundances) to reference abundances, including cosine similarity (dot product), Euclidean distance, absolute value distance, Manhattan distance, and Pearson correlation [9,10,11,12,13,14]. A common issue with these metrics is a high false discovery rate (FDRs), which is a quantity that describes the proportion of incorrect matches. These high FDRs are due in part to underutilizing peaks at larger *m*/*z* values that are still informative but have smaller abundances [9]. Early suggestions to address this problem encouraged manual selection of the correct compound from the top-n (generally 3–10) hits [11]; but, as untargeted compound identification pipelines increase their throughput, this has become an untenable solution.

**Figure 1:**
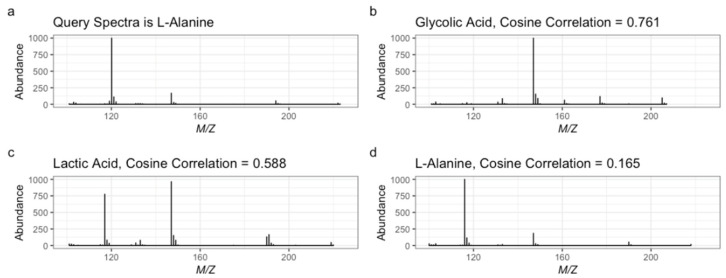
Example mass spectra displaying (**a**) the query spectrum and potential candidate spectra (**b**) glycolic acid, (**c**) lactic acid, and (**d**) alanine (the true match) with their cosine correlation score.

To combat high FDRs, newer metrics scale reference and measured abundance values by their respective *m*/*z*. This set of weighted metrics include composite similarity, weighted cosine similarity, and discrete wavelet (DWT) and Fourier transformation (DFT) [9,10,11]. These approaches require optimized parameters which vary based on query and reference library sizes [9,12,15]. Compounds with similar mass spectra but different structures also prove to be a significant challenge as the true match is not often the first ranked spectra (Figure 1) [15]. More recent deep learning approaches have also been developed that have higher accuracies than more traditional metrics [14,16]. However, traditional SS metrics still play a role in some deep learning approaches. For example, Hu et al. [16] use traditional metrics to first generate top-n lists of candidate spectral matches that are then fed into their deep learning classifier.

Despite these advances, there is little to no consensus on an optimum (highest accuracy) metric across multiple experiments [9,10,11,12,14,16,17,18], and the popularity of metrics also varies. Several metrics, for example, are either variations of or are benchmarked against cosine correlation [19]. This lack of standard practices in SS metric selections presents a reproducibility issue in metabolomics data processing wherein different sets of matches (i.e., identified metabolites) may be generated depending on the choice of similarity score. As the metabolomic content of a sample is fundamental to all subsequent inference involving those data, any biases introduced during this identification step would propagate in subsequent analyses. Within the context of One Health, this inconsistency in metric and any resultant misidentified metabolites may generate false understandings of the processes that underly the interactions between organism and environment, potentially leading to maligned interventions or system predictions.

Thus, to provide guidance towards reaching a standard identification workflow, we evaluate 66 similarity metrics for their ability to discriminate between true and false matches. These scores collectively represent ten different families of metrics, and the scores are evaluated using 4,521,216 hand-verified candidate spectra matches across multiple sample types including fungi (*Aspergillus niger*, *Aspergillus nidulans*, and *Trichoderma reesei*), soil crust, standards, standard mixtures, and human samples of cerebrospinal fluid (CSF), blood plasma, and urine. Through our investigation of these scores, we identify clusters of metrics that perform best in identifying true matches and characterize these clusters using their properties, such as their metric family, with a random forest model. This work represents the most comprehensive evaluation of similarity score metrics for GC-MS metabolite identification to date. Further, the analyses presented here provide a useful demonstration of the challenges in tasks as simple as comparing spectra that must be addressed to integrate metabolomic data—or any other data with limited consensus on assessment or reporting—in One Health workflows.

## 2. Materials and Methods

### 2.1. Data Acquisition

Sample acquisition, metabolite identification, and truth annotation are described elsewhere [20,21]. Briefly, samples collected from 2016 to 2020 underwent MS acquisition with an Agilent GC 7890A coupled with a single quadropole MSD 5975C (Agilent Technologies, Santa Clara, CA, USA) over a mass range of 50–550 *m*/*z*. Query spectra were matched to reference spectra for specific metabolites with the CoreMS software, version 1.0.0 [22]. True positives, true negatives, and unknowns were all manually verified with a qualified chemist using the Automated Spectral Deconvolution and Identification System (AMDIS) for all 4,521,216 spectra candidate matches. Specific rules to distinguish true negatives and unknowns are reported in [20].

### 2.2. Metrics and Metric Families

Numerous distance-based metrics have been developed to solve similarity problems [23]. Here, spectral intensities for the query and reference spectra were fully joined by *m*/*z* and missing intensities were denoted with a 0. Spectral intensities were either scaled by using the sum of measured intensities within a spectrum (i.e., sum or total intensity) or by using the spectrum’s maximum intensity (max). These scaled intensities are henceforth referred to as “abundances”. All metrics (Table S1) were coded by hand in Python 3.8.5 and families of similar metrics were defined based on their similar properties.

Ten families, or groups, of metrics were identified among the 66 metrics considered. These include the Chi Squared, Correlative, Fidelity, Inner Product, Intersection, $L_{1}$, $L_{p}$, Shannon’s Entropy, Vicis Wave Hedges, and Combined [11,23] families of metrics. Importantly, these families were defined according to Cha [23], which grouped metrics by their mathematical properties. These families are thus not definitive, and metrics may arguably fit under multiple families or be arranged into new families depending on user preference. For example, the $L_{p}$ family includes metrics that involve computation of the $L_{1}$-distances (i.e., |a−b|), yet there exist an entirely separate family of metrics based solely on $L_{1}$-distances (i.e., the $L_{1}$ family). Brief descriptions of each family are subsequently provided.

Chi Squared metrics all range from 0 to infinity (i.e., are left-bounded) and use the sum of squared difference between query and reference intensities (Table S1). Chi Squared-related scores are known to underperform at small sample sizes (number of peaks) [23]. Traditional correlation metrics (Pearson, Spearman, and Kendall Tau) range from –1 to 1, and perform best with linearly correlated data. Fidelity family metrics depend on geometric means and perform best for approximately normally distributed data [23]. All inner product metrics use the product of the query and reference spectra and are typically known to perform well for spectral matching [9,10]. The Intersection family utilizes either the minimum or maximum intensity per *m*/*z,* and thus tends to be sensitive to outliers [23]. $L_{1}$ metrics use the absolute difference between abundances and are typically sensitive to small differences [23]. The $L_{p}$ family includes Euclidean metrics that calculate the shortest distance between two points and is advantageous due to their simple design and implementation. Like $L_{1}$ metrics, these metrics are sensitive to small changes in values [23]. Shannon’s entropy metrics assume that an event (in this case, a present peak) is independent of other present peaks [23]. This assumption is typically violated for MS data where the presence of one peak may influence the presence of another [24]. The Vicis Wave Hedges family applies the properties of the Chi Squared family to the $L_{1}$ family metric Kulczynski distance, so it may underperform at small sample sizes and be sensitive to outliers. The remaining two metrics—average ($L_{1}$, $L_{\infty })$ divergence and the Taneja distance—do not fit cleanly under any one family due to their overlap between multiple families of metrics and are labeled as “Combination” metrics.

### 2.3. Pearson Correlation, T-Statistic, and Overlap Score

All statistical analyses have been made available at: https://github.com/PNNL-m-q/metabolomics_spectral_similarity_score (accessed on 4 May 2023). To determine metric relatedness, a Pearson correlation score was calculated between every pair of scores for both the sum and the max scaled abundance. Clusters were defined using complete agglomerative hierarchical clustering, such that highly correlated metrics were grouped together. This method was implemented, and its output visualized, using the pheatmap [25] package.

To understand the distributional differences of scores within clusters, a t-statistic and an overlap score were calculated. An unpaired, two-sided t-statistic was used to measure the difference in mean value between true positive and true negative distributions for each score. An overlap score was calculated as the proportion of true positives that overlap with the inner quartile ranges (between the 25th and 75th percentiles) of the true negative and unknown distributions over the total number of true positives.

### 2.4. Understanding Clusters of Similar Scores

To understand which score properties (such as the score’s family, bounds, etc.) within clusters contribute the most to the definition of that cluster, a random forest classification model was run using the ranger [26] package, and optimized using the tidy model implementations of the recipes [27], rsample [28], parsnip [29], workflowsets [30], yardstick [31], dials [32], vip [33], and tune [34] packages. The response variable was the specific cluster that a score belonged to, and the predictor variables were that cluster’s family (as defined in Section 2.2), type (i.e., whether a distance-based or similarity-based metric), theoretical bound direction (i.e., left-bound, right-bound, both, or none), basic distributional properties (min, median, and max score), and its t-statistic and overlap scores (as defined in Section 2.3).

Data were split into 75% training (50 scores) and 25% testing (16 scores), and class balance was checked to be relatively equal between the training and testing datasets. To determine optimum random forest parameters, cross validation with 10% holdout was conducted 5 times using the training data, and the parameter set associated with the highest model accuracy was selected. Using the testing data, we then compared predicted classifications from the optimized random forest model to the true classifications. The relative importance of each variable in the random forest model was determined using the Gini Index [35]. Random forest models were grown for both the sum and max scaled datasets.

## 3. Results

**Figure 2:**
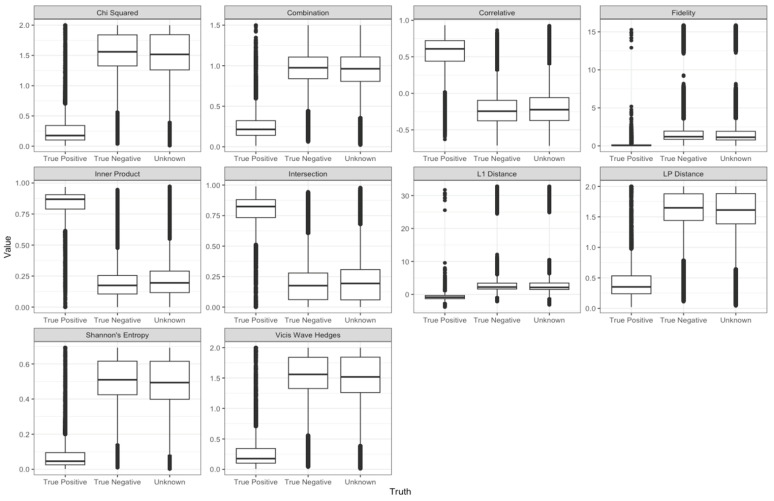
Representative distributions of scores from each family.

For both sum and max normalized variants of spectra, we visualized the distributions of true positive (i.e., correct identification), true negative (i.e., correct non-identification), and unknown annotations for each metric. The full set of these figures are presented within an interactive trelliscope [36] display accessible at the following link: https://pnnl-m-q.github.io/metabolomics_spectral_similarity_score/ (accessed on 29 August 2023). However, here, examples of these distributions are provided from each of the ten families of metrics considered (Figure 2).

Across the distributions displayed in Figure 2, each indicate clear differences between true positive and the non-true positive (true negative and unknown) distributions, but the degree to which these distributions differ varies across metrics. Taking the distributions shown from the Chi Squared and Combination families as examples, we see that both have true positive distributions centered between 0 and 0.5, but the Chi Squared metric has true negative and unknown distributions centered near 1.5, whereas the Combination metric indicates these distributions are centered near 1. Thus, the displayed example from the Chi Squared metric better separates true positives from others relative to the Combination metric example since the difference in distributional centers is greater. A more formal way of quantifying this visual comparison is through the computation of t-statistics, which measures the difference in the means of each distribution while accounting for their variability. We additionally computed ‘overlap scores’ that measure the proportion of true positive scores whose values coincide with those of the true negative and unknown distributions. We used these statistics as the basis for evaluating how well scores are able to distinguish between true positive and non-true-positive matches. Prior to this evaluation, however, we were interested in quantifying the degree of shared information across metrics and whether metrics that capture similar information map to the same metric families.

### 3.1. Correlated Metric Groups

**Figure 3:**
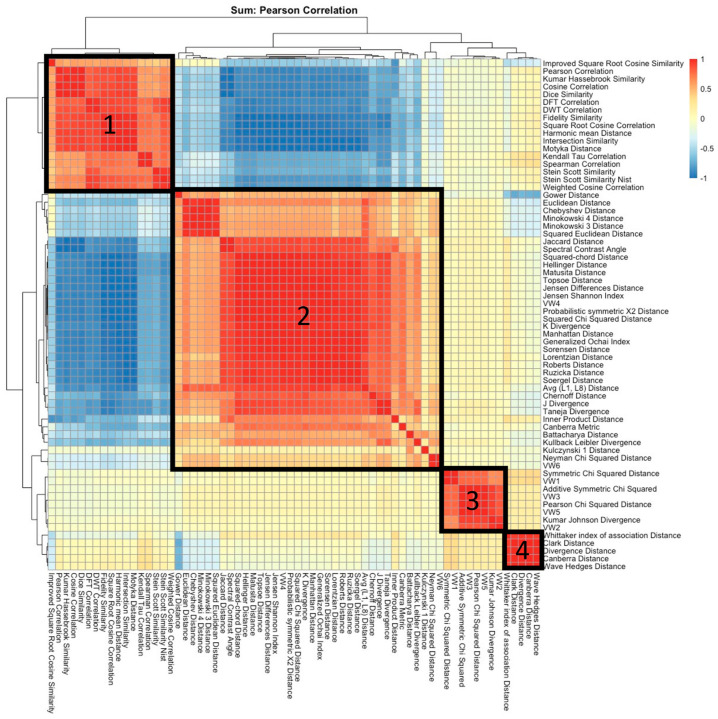
Heatmap of Pearson correlations measured between each pair of similarity metric, where each score is computed based on the sum normalized data. Numbered boxes denote clusters of high positively correlated metrics.

Using agglomerative hierarchical clustering of Pearson correlations between metrics, four clusters were defined for the sum normalized data (Figure 3). A similar figure based on the max normalized data is available in the supplement (Figure S1). Metrics in this heatmap are clustered to reveal four visually distinctive groups of metrics, each labeled in the heatmap. From this heatmap, we see that Clusters 1 and 2 are mostly negatively correlated, as indicated by the blue shading in the off-diagonals between them. The negative correlation between these groups of metrics suggests that these two groups contain similar information but quantify this information in opposing “directions”: Cluster 1 largely consists of similarity metrics that *increase* in value the more similar two spectra are to one another, whereas Cluster 2 largely consists of “distance” metrics that *decrease* in value the more similar (i.e., less distant) two spectra are to one another. On the other hand, Cluster groups 3 and 4 appear to share little to no information between themselves and other cluster groups given that metrics in these groups indicate high positive correlations only within their own groups and near-zero correlation otherwise. The observed groupings in Figure 3 based on the sum normalized data are consistent with those yielded by the max-normalized data.

**Figure 4:**
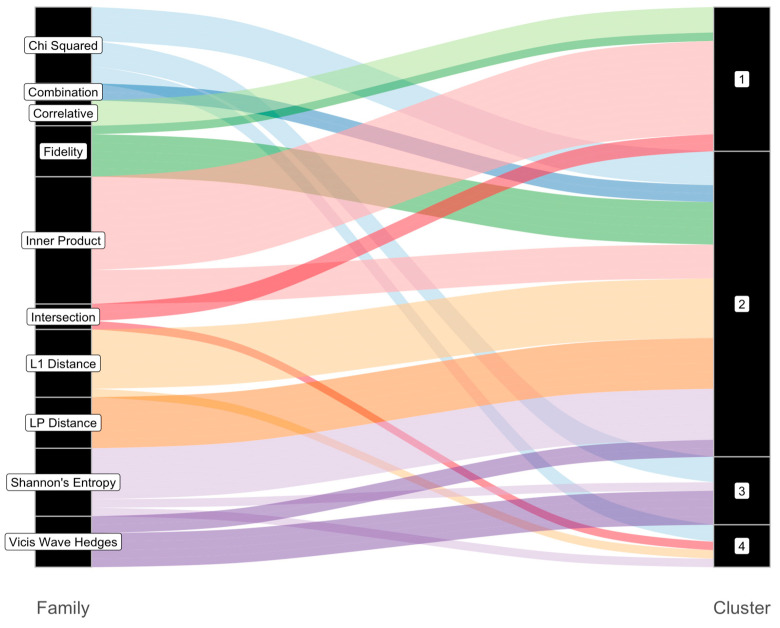
Alluvial plot indicating how metric family groups (**left**) map to Pearson correlation clusters (**right**).

The metrics considered in our analyses are from ten mathematical families of metrics; however, empirically, Figure 3 demonstrates that these metrics cluster into four groups. Figure 4 indicates that each family maps to more than one of the four empirical cluster groups, yet some families represent most metrics within a given cluster group. The first empirical cluster group, shown on the right of Figure 4 and labeled as “1”, largely consists of metrics from the Inner Product family; empirical cluster group “2” contains all the $L_{p}$ family metrics, but these represent a similar proportion of cluster group “2” metrics as the Chi Squared, Fidelity, $L_{1}$, and Shannon’s entropy families; cluster group “3” mostly contains metrics from either the Chi Squared or Vicis Wave Hedges families; and cluster group “4” represents metrics from the Chi Squared, Intersection, $L_{1}$, and Shannon’s entropy families in roughly equal proportion.

**Figure 5:**
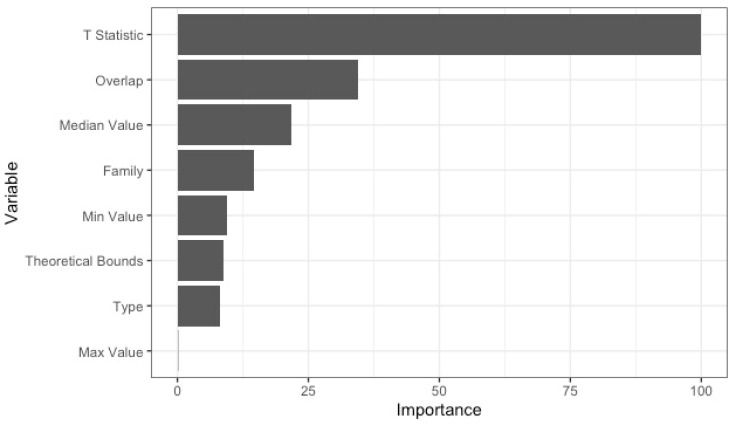
Variable importance plot displaying the importance of variables within a random forest model fitted to sum abundance data to predict cluster membership. Values are scaled such that importance varies between 0 and 100, with a value of 100 indicating the most important variable.

Given that metric family alone does not perfectly map to each of the four empirical cluster groups, we fit random forest models to determine which metric attributes best predict cluster membership. The optimized random forest model consisted of 1395 decision trees formed by sampling from three variables at each split. On the test dataset of 16 metrics, the model had 100% accuracy. The importance of each attribute is shown in Figure 5, scaled such that a value of 100 indicates the greatest level of importance. Among all variables considered, the computed t-statistics of metrics is definitively the most important in dictating cluster group membership. Since, in this context, the t-statistic measures how different the true positive and non-true-positive distributions are from one another for each metric, the high importance of the t-statistic indicates that the four empirical cluster groups are largely determined by the discriminatory ability of each metric. This finding is further validated by the fact that the second-most important factor for predicting group membership is the overlap score, which is an alternative measure for how different true and non-true positive distributions are. The results shown in Figure 5 are based on a random forest model fitted to the sum abundance data; the ranking of variables from most important to least based on the max abundance data are identical to the sum abundance rankings and are included in the supplement.

### 3.2. Metric Performance

Having defined four groups of metrics and determining which score factors are predictive of said groups, we next characterized each of these groups based on their average values for the top three most important predictive factors for group membership. These values are provided in Table 1.

Clusters 1 and 2 have the largest magnitude t-statistics and smallest magnitude overlap score. Together, these results indicate that these two cluster groups contain metrics that—on average—better discriminate between true and non-true positives. The high magnitude t-statistics suggest that the means of the true and non-true positive distributions resulting from these metrics are further apart from one another, even after accounting for the variability of each distribution. For additional context, an absolute t-statistic value larger than 1.96 would yield a significant *p*-value at the 0.05 level in a traditional comparison of means. Here, the t-statistics were more than 200 times this threshold value. Interestingly, the absolute t-statistic for cluster 1 and 2 are very similar, which supports the evidence provided by the hierarchical clusters (Figure 3) that cluster groups 1 and 2 are similarly discriminated but in opposing directions. Scores in cluster 1 have a higher score for true positives over true negatives, and the opposite was true in cluster 2. High average magnitudes for t-statistics are also observed for cluster groups 3 and 4, but their average overlap scores suggest as much as seven times the degree of distributional overlap as cluster groups 1 and 2: whereas cluster groups 1 and 2 are associated with an average overlap between 2 and 3%, and cluster groups 3 and 4 have overlaps of ~14% and ~21%, respectively.

## 4. Discussion

The analyses presented within this work are meant to provide researchers with better guidance towards which of the multitude of metrics may most reliably be used to identify true GC-MS matches from among several candidate compounds. We have found that different collections of metrics quantify similarity in related ways, and thus there is not one “best” metric; instead, there are well-performing *groups* of metrics. Of the ten mathematical families of metrics we investigated, the Inner Product, Correlative, and Intersection families were those that were most dominantly represented across both best-performing empirical cluster groups. This considered, it is important to note that within each of these cluster groups, there exists variability in metric performance. Though, on average, cluster groups 3 and 4 did not discriminate between true and non-true positives as well as cluster groups 1 and 2, there exists metrics within these lesser-performing cluster groups that may indicate better distributional characteristics (in terms of true positive discrimination) relative to some metrics within the better performing groups. For example, the Whittaker Index of Association Distance is in cluster 4 but has a t-statistic of −354, which was more than twice the average t-statistic (Table 1) in cluster 4. In a more general example showcasing the variability of metric performance, we compare the Stein Scott Similarity, Cosine Correlation, and Canberra Metric (Figure S2). In practice, a score threshold based on the experimental data and analyst experience is applied to separate true positives and negatives. The comparison depicted in Figure S2 shows how true positive and true negative proportions change for two commonly used scores (Stein Scott and Cosine Correlation) and one moderately performing score (Canberra Metric). Though Cosine Correlation tends to have the highest proportion of true positives at higher score thresholds, it also has the highest proportions of true negatives (Figure S2). The NIST implementation of Stein Scott Similarity does not appear to have the highest proportion of true positives at higher score thresholds, but it does have lower proportions of true negatives. The comparison depicted in Figure S2 demonstrates that the choice of score and threshold will affect the number of true positives and true negatives that are matched to query spectra, with some scores being better able to “filter out” true negatives at different thresholds than others. The previous two examples serve to emphasize the fact that there are differences in metric performance, with many—not just a select few—that perform well. This fact then motivates a direction for future advancement in similarity score research, wherein one may consider the use of an ensemble of metrics in predicting whether a match is a true positive. Ensemble approaches to modeling have been known to exhibit improved predictive performance over their non-ensemble counterparts [37], and thus developing a similarity score based on an ensemble of others may yield improved performance over any of its individual contributors.

Metrics that assume linearity (e.g., Spearman Correlation, Kendall Tau Correlation, etc.) and are sensitive to small differences in abundance values (e.g., Kulczynski 1 Distance, Canberra Metric) and outliers (e.g., Intersection Similarity, Motyka Distance) tended to land in the first and second cluster of metrics with higher absolute t-statistics and lower overlap scores on average, which may suggest that these assumptions are valid for abundances (scaled intensities). Within this dataset, peaks with high abundances tended to have higher *m*/*z* values which may be linearly correlated. The scaling may also reduce large outliers and large differences in intensities. Clusters 1 and 2 tended to include metrics that are reported to have high performances in separating true positives and true negatives, including Stein Scott Similarity, DFT/DWT correlation, and Weighted Cosine Correlation [9]. Metrics that assume independent peaks (e.g., Kumar Johnson Divergence) and large data sizes (e.g., VW1, VW2) tended to land in the third and fourth cluster of metrics with lower absolute t-statistics and high overlap scores on average, which suggests that these assumptions are suboptimal when selecting SS metrics.

While not included among the primary results of this work, our analyses revealed trends that suggest improved discrimination between true and false positives across all metrics when using the sum approach over the max approach to normalization. For example, the average overlap score for cluster 2 in the sum dataset was 0.026 (Table 1) and 0.034 in the max scaled dataset (Table S2). Though a marginal difference, this does suggest some improvement when using sum scaling over max scaling since fewer of the true positive match scores overlapped in distribution with the scores of the true negative/unknown candidate matches. Last, a common problem in annotating metabolite spectra is the presence of unknowns, given that so much of the metabolome has yet to be fully characterized [38]. Our results show that the similarity scores of unknown annotations have distributions that are largely like those of true negative annotations. These “unknown” annotations typically represent cases where experts are unwilling to rule out the candidate as a possible match, but do not have enough evidence to definitively label it as a non-match (i.e., true negative). Thus, as we show that the true negative and unknown score distributions are largely similar across all metrics, labeling “unknowns” as true negatives may not necessarily bias any inference involving comparisons between these and the true positive score distributions. For example, if one were interested in obtaining the false discovery rate (FDR) for a given metric applied to labeled data, one would need to know the proportion of true negatives among all the matches made using the similarity metric of interest. Matches labeled as “unknown” are problematic in this context since it is unclear whether these matches should be counted as correct (i.e., a true positive) or as an erroneously matched true negative. However, the findings of this work suggest that it would be reasonable to treat these unknown annotations as true negatives.

The discussion of unknown annotations leads to important limitations of the present work. A large majority of the metabolome has yet to be discovered, and thus there is an overabundance of unknowns across our samples. This leaves room for uncertainty regarding the true performance of each metric considering that the score distribution for unknown samples may represent a mixture of true positive and true negative matches. However, this limitation has been partially compensated for by our large volume of samples and the high amounts of non-unknown annotations in each. Beyond the presence of unknown annotations, it is also worth nothing that our data were manually annotated by chemists and thus are subject to some degree of misclassification error.

## 5. Conclusions

Our findings do not point to any one metric as a clear optimum, yet we find that there are clear groups of metrics that tend to perform well relative to others. Within the best performing metric cluster (cluster 1), we found that the NIST implementation of Stein Scott Similarity [9] and the DFT correlation metrics both appeared within the top three of ranked lists generated based on overlap score and the T-statistic. We therefore recommend the use of either of these metrics, or metrics from either of the better performing groups (i.e., clusters 1 and 2). More generally, we encourage readers to rely on the t-statistics and overlap scores reported in this work to identify and select well-performing metrics: the best performing metrics are those with higher t-statistics and lower overlap scores, as this jointly indicates that the corresponding metric assigns different values to true positives than it does to non-true positives. All these metrics may be implemented through the CoreMS software [22]. We caution the reader against the belief that these recommended metrics are guaranteed to best identify correct matches across all samples. As discussed earlier, metrics from other clusters have merit and may exhibit an outperformance in some cases.

There is, thus, a nuance to selecting spectral similarity metrics within metabolomic identification pipelines. Each data stream within a One Health data science workflow—human, veterinary, plant, and environment—will have different nuances that either need to be addressed or made consistent in order to best utilize those data streams in an integrated approach. It is clear from the SS analyses presented here that there is a great benefit to improving our understanding of current standard analytical practices and how to make them more consistent and standardized to minimize any error across a more holistic One Health analytical pipeline. We hope through this work to start a discussion of these nuances across data streams and data scales to build more robust data science approaches to One Health.

## Figures and Tables

**Table 1:** Table of the average values for each of the three most important factors in predicting cluster membership for the sum scaled dataset.

Cluster	t-Statistic	Overlap Score	Score Median *
1	482.798	0.022	0.120
2	−412.002	0.026	6.63 × 10^10^
3	−47.049	0.139	2.513 × 10^29^
4	−151.720	0.213	106.002

* column represents the average of the computed medians across metric distributions within this group.

## Data Availability

Data are available at https://doi.org/10.25584/PNNL.data/1902325.

## Supplementary Materials

The following supporting information can be downloaded at: https://www.mdpi.com/article/10.3390/metabo13101101/s1, Figure S1: Heatmap of Pearson correlations measured between each pair of similarity metric, where each score is computed based on the max normalized data; Table S1: The tested 66 metrics with their cluster number, their overlap score, their respective family, their ranges, and formula; Table S2: Average values for each of the three most important factors in predicting cluster membership for the max scaled dataset; Figure S2: Proportion of true positives or true negatives with Canberra Metric score, Cosine Correlation, or NIST Stein Scott Similarity score above the indicated threshold, relative to the combined total of true positives and negatives above the threshold. Refs. [8,10] are cited in Supplementary Materials.

## References

[1] Gibbs E.P.J. (2014). The evolution of One Health: A decade of progress and challenges for the future. Vet. Rec..

[2] Pećina-Šlaus N., Pećina M. (2015). Only one health, and so many omics. Cancer Cell Int..

[3] Manrai A.K., Cui Y., Bushel P.R., Hall M., Karakitsios S., Mattingly C.J., Ritchie M., Schmitt C., Sarigiannis D.A., Thomas D.C. (2017). Informatics and Data Analytics to Support Exposome-Based Discovery for Public Health. Annu. Rev. Public Health.

[4] Traversi D., Ripabelli G. (2022). Editorial: New omics research challenges for Public and sustainable Health. Front. Microbiol..

[5] Tigistu-Sahle F., Mekuria Z.H., Satoskar A.R., Sales G.F.C., Gebreyes W.A., Oliveira C.J.B. (2023). Challenges and opportunities of molecular epidemiology: Using omics to address complex One Health issues in tropical settings. Front. Trop. Dis..

[6] Cabal A., Martinovic A. (2022). Special Issue ‘One Health meets Omics: The way forward to investigate zoonosis’. J. Appl. Microbiol..

[7] Hajjar G., Barros Santos M.C., Bertrand-Michel J., Canlet C., Castelli F., Creusot N., Dechaumet S., Diémé B., Giacomoni F., Giraudeau P. (2023). Scaling-up metabolomics: Current state and perspectives. TrAC Trends Anal. Chem..

[8] Hotea I., Sirbu C., Plotuna A.M., Tîrziu E., Badea C., Berbecea A., Dragomirescu M., Radulov I. (2023). Integrating (Nutri-)Metabolomics into the One Health Tendency—The Key for Personalized Medicine Advancement. Metabolites.

[9] Kim S., Koo I., Jeong J., Wu S.W., Shi X., Zhang X. (2012). Compound Identification Using Partial and Semipartial Correlations for Gas Chromatography-Mass Spectrometry Data. Anal. Chem..

[10] Koo I., Zhang X., Kim S. (2011). Wavelet- and Fourier-transform-based spectrum similarity approaches to compound identification in gas chromatography/mass spectrometry. Anal. Chem..

[11] Stein S.E., Scott D.R. (1994). Optimization and testing of mass spectral library search algorithms for compound identification. J. Am. Soc. Mass Spectrom..

[12] Koo I., Kim S., Zhang X. (2013). Comparative analysis of mass spectral matching-based compound identification in gas chromatography-mass spectrometry. J. Chromatogr. A.

[13] Kim S.H., Ouyang M., Jeong J., Shen C.Y., Zhang X. (2014). A New Method of Peak Detection for Analysis of Comprehensive Two-Dimensional Gas Chromatography Mass Spectrometry Data. Ann. Appl. Stat..

[14] Matyushin D.D., Sholokhova A.Y., Buryak A.K. (2020). Deep Learning Driven GC-MS Library Search and Its Application for Metabolomics. Anal. Chem..

[15] Kim S., Zhang X. (2015). Discovery of false identification using similarity difference in GC-MS-based metabolomics. J. Chemometr..

[16] Hu Q., Zhang J., Chen P., Wang B. (2021). Compound identification via deep classification model for electron- ionization mass spectrometry. Int. J. Mass Spectrom..

[17] Zhang J., Xia Y., Zheng C.H., Wang B., Zhang X., Chen P. (2016). Combine multiple mass spectral similarity measures for compound identification. Int. J. Data Min. Bioin..

[18] Wei X.L., Koo I., Kim S., Zhang X. (2014). Compound identification in GC-MS by simultaneously evaluating the mass spectrum and retention index. Analyst.

[19] Scheubert K., Hufsky F., Böcker S. (2013). Computational mass spectrometry for small molecules. J. Cheminform..

[20] Degnan D.J., Bramer L.M., Flores J.E., Paurus V.L., Corilo Y.E., Clendinen C.S. (2023). Evaluating Retention Index Score Assumptions to Refine GC–MS Metabolite Identification. Anal. Chem..

[21] Flores J.E., Bramer L.M., Degnan D.J., Paurus V.L., Corilo Y.E., Clendinen C.S. (2023). Gaussian Mixture Modeling Extensions for Improved False Discovery Rate Estimation in GC–MS Metabolomics. J. Am. Soc. Mass Spectrom..

[22] Corilo Y.E., Kew W.R., McCue L. (2021). EMSL-Computing/CoreMS: CoreMS 1.0.0.

[23] Cha S.H. (2007). Comprehensive survey on distance/similarity measures between probability density functions. Int. J. Math. Model Meth. Appl. Sci..

[24] Vaniya A., Fiehn O. (2015). Using fragmentation trees and mass spectral trees for identifying unknown compounds in metabolomics. Trends Analyt. Chem..

[25] Kolde R. (2019). Pheatmap: Pretty Heatmaps.

[26] Wright M.N., Ziegler A. (2017). Ranger: A Fast Implementation of Random Forests for High Dimensional Data in C++ and R. J. Stat. Softw..

[27] Kuhn M., Wickham H., Hvitfeldt E. (2023). Recipes: Preprocessing and Feature Engineering Steps for Modeling.

[28] Frick H., Chow F., Kuhn M., Mahoney M., Silge J., Wickham H. (2022). Rsample: General Resampling Infrastructure.

[29] Kuhn M., Vaughan D. (2023). Parsnip: A Common API to Modeling and Analysis Functions.

[30] Kuhn M., Couch S. (2023). Workflowsets: Create a Collection of ‘Tidymodels’ Workflows.

[31] Kuhn M., Vaughan D., Hvitfeldt E. (2023). Yardstick: Tidy Characterizations of Model Performance.

[32] Kuhn M., Frick H. (2023). Dials: Tools for Creating Tuning Parameter Values.

[33] Greenwell B.M., Boehmke B.C. (2020). Variable Importance Plots—An Introduction to the vip Package. R J..

[34] Kuhn M. (2023). Tune: Tidy Tuning Tools.

[35] Ceriani L., Verme P. (2012). The origins of the Gini index: Extracts from Variabilità e Mutabilità (1912) by Corrado Gini. J. Econ. Inequal..

[36] Hafen R., Schloerke B. (2021). Trelliscopejs: Create Interactive Trelliscope Displays.

[37] Sagi O., Rokach L. (2018). Ensemble learning: A survey. Wiley Interdiscip. Rev. Data Min. Knowl. Discov..

[38] da Silva R.R., Dorrestein P.C., Quinn R.A. (2015). Illuminating the dark matter in metabolomics. Proc. Natl. Acad. Sci. USA.

