# Supplementary material for: Characterizing Families of Spectral Similarity Scores and Their Use Cases for Gas Chromatography–Mass Spectrometry Small Molecule Identification
Source: Metabolites. 2023 Oct 21;13(10):1101. doi: 10.3390/metabo13101101 (PMC10608912; doi:10.3390/metabo13101101)
Supplement: Supplementary file 1 [file metabolites-13-01101-s001.zip › metabolites-2637135-supplementary.pdf]

Supporting Information for

Characterizing Families of Spectral Similarity Scores and Their Use Cases for Gas Chromatography–Mass Spectrometry Small Molecule Identification

David J. Degnan [1,†], Javier E. Flores [1,†], Eva R. Brayfindley [2], Vanessa L. Paurus [3], Bobbie-Jo M. Webb-Robertson [1], Chaevien S. Clendinen [3] and Lisa M. Bramer [1,*]

1 Biological Sciences Division, Pacific Northwest National Laboratory, Richland, WA 99354, USA; david.degnan@pnnl.gov (D.J.D.); javier.flores@pnnl.gov (J.E.F.); bj@pnnl.gov (B.-J.M.W.-R.)

2 Artificial Intelligence and Data Analytics Division, Pacific Northwest National Laboratory, Richland, WA 99354, USA; eva.brayfindley@pnnl.gov

3 Environmental and Molecular Sciences Division, Pacific Northwest National Laboratory, Richland, WA 99354, USA; vanessa.paurus@pnnl.gov (V.L.P.); chaevien.clendinen@pnnl.gov (C.S.C.)

* Correspondence: lisa.bramer@pnnl.gov

† These authors contributed equally to this work.

Table of Contents

**Figure S1**	Heatmap of Pearson correlations measured between each pair of similarity metric, where each score is computed based on the max normalized data.	Page S2
**Table S1**	The tested 66 metrics with their cluster number, their overlap score, their respective family, range, and formula.	Page S3
**Table S2**	Average values for each of the three most important factors in predicting cluster membership for the max scaled dataset.	Page S7
**Figure S2**	Proportion of true positives (left) or true negatives (right) with Canberra Metric score (red), Cosine Correlation (green), or NIST Stein Scott Similarity (blue) score above the indicated threshold, relative to the combined total of true positives and negatives above the threshold.	Page S8

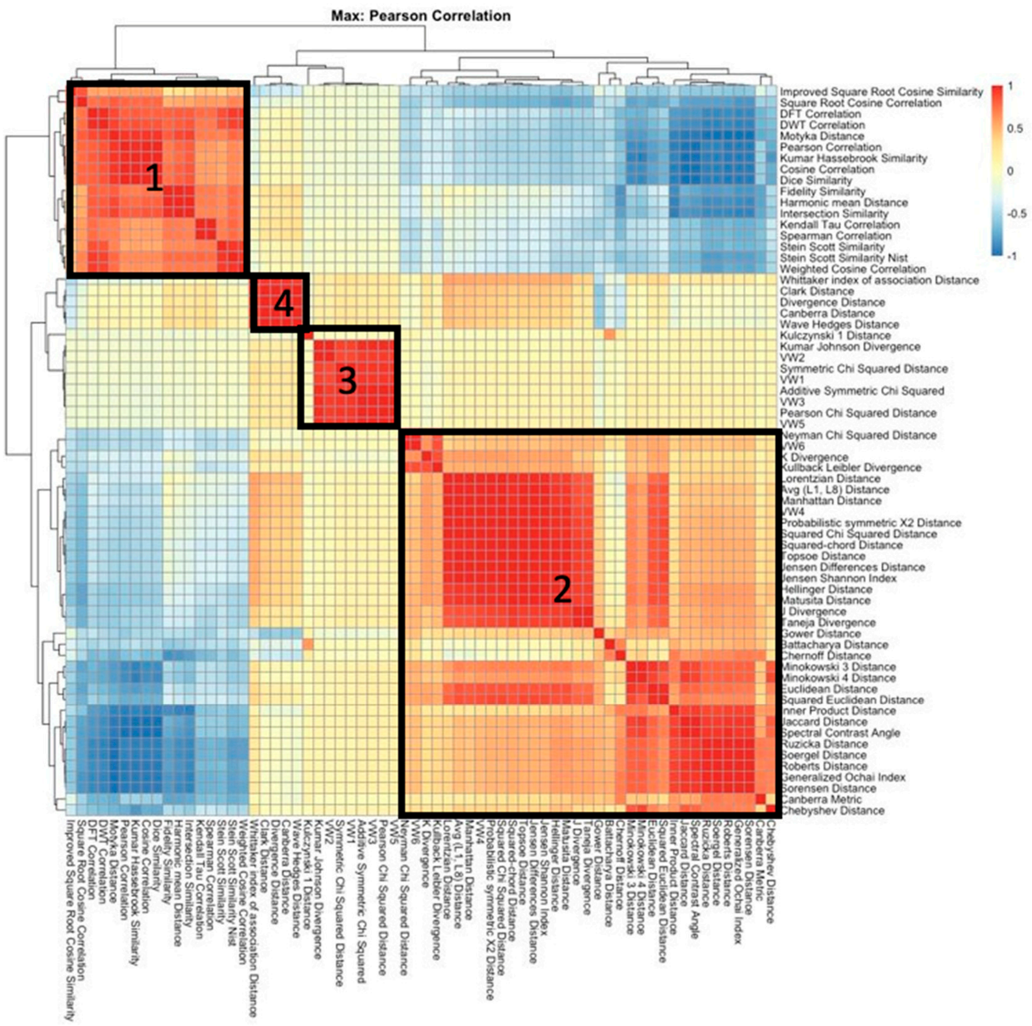

Figure S1. Heatmap of Pearson correlations measured between each pair of similarity metric, where each score is computed based on the max normalized data. Numbered boxes denote clusters of high positively-correlated metrics. The metrics within these clusters are the same as in the sum normalized data.

Table S1. The tested 66 metrics with their cluster number, their overlap score, their respective family, and formula. In the formula, for a given metabolite spectra, let X and Y denote vectors, of length N, representing m/z peak locations and peak intensities, respectively. Then, x_i denotes the m/z location and y_i denotes the peak intensity for the ith peak for the reference spectrum and $i = 1, 2, I, N$. Similarly, let U and V denote vectors, of length N, representing m/z peak locations and peak intensities for a query spectrum. Thus, u_i denotes the observed m/z peak location for the ith peak of the query spectrum, such that $|x_i - u_i| \leq 0.5$, and v_i denotes the observed peak intensity for the ith peak. For brevity, we define $\sum_i = \sum_{i=1}^{N}$

Metric Name	Cluster	Overlap Score	T-Statistic	Family / Range	Formula
Stein Scott Similarity NIST [8]	1	0.01201	560	Inner Product / [0,1]	$\frac{[\sum_i I(v_i \neq 0)]S_{wc} + [\sum_i I(v_i \neq 0) I(y_i \neq 0)]S_r}{\sum_i I(v_i \neq 0) + \sum_i I(v_i \neq 0) I(y_i \neq 0)}$, $S_r = \frac{\sum_{\{i\|v_i \neq 0,\, y_i \neq 0\}} \left(\frac{y_i}{y_{i-1}} * \frac{v_{i-1}}{v_i}\right)^n}{\sum_i I(v_i \neq 0) I(y_i \neq 0)}$, $n = \begin{cases} 1 & if \left(\frac{y_i}{y_{i-1}} * \frac{v_{i-1}}{v_i}\right) < 1 \\ -1 & if \left(\frac{y_i}{y_{i-1}} * \frac{v_{i-1}}{v_i}\right) \geq 1 \end{cases}$ S_{wc} is Weighted Cosine Correlation with $a = 0.5, b = 1.3$
Stein Scott Similarity [10]	1	0.01298	515	Inner Product / [0, 1]	$\frac{[\sum_i I(v_i \neq 0)]S_{wc} + [\sum_i I(v_i \neq 0) I(y_i \neq 0)]S_r}{\sum_i I(v_i \neq 0) + \sum_i I(v_i \neq 0) I(y_i \neq 0)}$, $S_r = \frac{\sum_{\{i\|v_i \neq 0,\, y_i \neq 0\}} \left(\frac{y_i}{y_{i-1}} * \frac{v_{i-1}}{v_i}\right)^n}{\sum_i I(v_i \neq 0) I(y_i \neq 0)}$, $n = \begin{cases} 1 & if \left(\frac{y_i}{y_{i-1}} * \frac{v_{i-1}}{v_i}\right) < 1 \\ -1 & if \left(\frac{y_i}{y_{i-1}} * \frac{v_{i-1}}{v_i}\right) \geq 1 \end{cases}$ S_{wc} is Weighted Cosine Correlation with $a = 0.6, b = 3$
DFT Correlation	1	0.01305	579	Inner Product / [-1, 1]	$\frac{N_V S_w(y_p, v_p) + N_{V \wedge Y} S_{DFT}(y_p, v_p)}{N_V + N_{V \wedge Y}}$ $S_{DFT}(y_p, v_p) = \frac{\sum_P y_{DFT} v_{DFT}}{(\sum_P y_{DFT}{}^2)^{1/2} (\sum_P v_{DFT}{}^2)^{1/2}}$
Harmonic Mean Distance	1	0.01327	529	Inner Product / [0, Inf)	$2 \sum_i \frac{y_i v_i}{y_i + v_i}$
Intersection Similarity	1	0.01364	457	Intersection [0, Inf)	$\sum_i min\{y_i, v_i\}$
Motyka Distance	1	0.01364	457	Intersection / [0, 0.5]	$\frac{\sum_i min\{y_i, v_i\}}{\sum_i (y_i + v_i)}$
DWT Correlation	1	0.01394	565	Inner Product / [-1, 1]	$\frac{N_V S_w(y_p, v_p) + N_{V \wedge Y} S_{DWT}(y_p, v_p)}{N_V + N_{V \wedge Y}}$ $S_{DWT}(y_p, v_p) = \frac{\sum_P y_{DWT} v_{DWT}}{(\sum_P y_{DWT}{}^2)^{1/2} (\sum_P v_{DWT}{}^2)^{1/2}}$
Fidelity Similarity	1	0.01394	549	Fidelity / [0, Inf)	$\sum_i \sqrt{y_i v_i}$
Square Root Cosine Correlation	1	0.01394	549	Inner Product / [0, 1]	$\frac{\sqrt{\sum_i y_i v_i}}{\sum_i y_i \sum_i v_i}$

Weighted Cosine Correlation	1	0.01439	557	Inner Product / [0, 1]	$S_{wc} = \frac{\sum_i (x_i{}^a y_i{}^b)(u_i{}^a v_i{}^b)}{(\sum_i (x_i{}^a y_i{}^b)^2)^{1/2} (\sum_i (u_i{}^a v_i{}^b)^2)^{1/2}}$
Kendall Tau Correlation	1	0.02262	386	Correlative / [-1, 1]	$\frac{2}{N(N-1)} \sum_{i<j} sgn(y_{i-} - y_j) sgn(v_{i-} - v_j)$
Spearman Correlation	1	0.02269	409	Correlative / [-1, 1]	Pearson correlation computed on ranked vectors
Dice Similarity	1	0.02410	499	Inner Product / [0, 1]	$\frac{2 \sum_i y_i v_i}{\sum_i y_i{}^2 + \sum_i v_i{}^2}$
Kumar Hassebrook Similarity	1	0.02410	424	Inner Product / [0, 1]	$\frac{\sum_i y_i v_i}{\sum_i y_i{}^2 + \sum_i v_i{}^2 - \sum_i y_i v_i}$
Cosine Correlation	1	0.02477	512	Inner Product / [0, 1]	$\frac{\sum_i y_i v_i}{(\sum_i y_i{}^2)^{1/2} (\sum_i v_i{}^2)^{1/2}}$
Pearson Correlation	1	0.02499	513	Correlative / [-1, 1]	$\frac{\sum_i (y_i - \bar{y})(v_i - \bar{v})}{\sqrt{\sum_i (y_i - \bar{y})^2 \sum_i (v_i - \bar{v})^2}}$
Improved Square Root Cosine Similarity	1	0.09039	150	Inner Product / [0, 1]	$\frac{\sqrt{\sum_i y_i v_i}}{\sqrt{\sum_i y_i} * \sqrt{\sum_i v_i}}$
VW4	2	0.01312	-509	Vicis Wave Hedges/ [0, Inf)	$\sum_i \frac{(y_i - v_i)^2}{\max(y_i, v_i)}$
Probabilistic Symmetric Chi-Squared Distance	2	0.01327	-529	Chi Squared / [0, Inf)	$2 \sum_i \frac{(y_i - v_i)^2}{y_i + v_i}$
Squared Chi Squared Distance	2	0.01327	-529	Chi Squared / [0, Inf)	$\sum_i \frac{(y_i - v_i)^2}{y_i + v_i}$
Jensen Differences Distance	2	0.01350	-540	Shannon's Entropy/ [0, Inf)	$\sum_i \left[\frac{y_i \ln y_i + v_i \ln v_i}{2} - \left(\frac{y_i + v_i}{2}\right) \ln\left(\frac{y_i + v_i}{2}\right)\right]$
Jensen Shannon Index	2	0.01350	-540	Shannon's Entropy/ [0, Inf)	$\frac{1}{2}\left[\sum_i y_i \ln\left(\frac{2y_i}{y_i + v_i}\right) + \sum_i v_i \ln\left(\frac{2v_i}{y_i + v_i}\right)\right]$
K Divergence	2	0.01350	-499	Shannon's Entropy/ [0, Inf)	$\sum_i v_i \ln\left(\frac{2v_i}{y_i + v_i}\right)$
Topsoe Distance	2	0.01350	-540	Shannon's Entropy/ [0, Inf)	$\sum_i \left[y_i \ln\left(\frac{2y_i}{y_i + v_i}\right) + v_i \ln\left(\frac{2v_i}{y_i + v_i}\right)\right]$
Generalized Ochai Index	2	0.01364	-457	Inner Product / [0, 1]	$1 - \frac{\sum_i \min\{y_i, v_i\}}{\sqrt{\sum_i y_i \sum_i v_i}}$
Kulczynski 1 Distance	2	0.01364	-25	L1 Distance / [0, Inf)	$\frac{\sum_i \lvert y_i - v_i \rvert}{\sum_i min\{y_i, v_i\}}$
Manhattan Distance	2	0.01364	-457	LP Distance / [0, Inf)	$\sum_i \lvert y_i - v_i \rvert$
Ruzicka Distance	2	0.01364	-373	L1 Distance / [0, 1]	$1 - \frac{\sum_i \lvert y_i - v_i \rvert}{\sum_i max\{y_i, v_i\}}$
Soergel Distance	2	0.01364	-373	L1 Distance / [0, 1]	$\frac{\sum_i \lvert y_i - v_i \rvert}{\sum_i max\{y_i, v_i\}}$
Sorensen Distance	2	0.01364	-457	LP Distance / [0, 1]	$\frac{\sum_i \lvert y_i - v_i \rvert}{\sum_i (y_i + v_i)}$
Battacharya Distance	2	0.01394	-409	Fidelity / (-Inf, Inf)	$-\ln\left(\sum_i \sqrt{y_i v_i}\right)$

Hellinger Distance	2	0.01394	-425	Fidelity / [0, Inf)	$\sqrt{2\sum_i\left(\sqrt{y_i}-\sqrt{v_i}\right)^2}$
Matusita Distance	2	0.01394	-425	Fidelity / [0, Inf)	$\sqrt{\sum_i\left(\sqrt{y_i}-\sqrt{v_i}\right)^2}$
Squared-Chord Distance	2	0.01394	-549	Fidelity / [0, Inf)	$\sum_i\left(\sqrt{y_i}-\sqrt{v_i}\right)^2$
Lorentzian Distance	2	0.01401	-456	L1 Distance / [0, Inf)	$\sum_i \ln(1+\lvert y_i - v_i\rvert)$
Roberts Distance	2	0.01490	-398	L1 Distance / [0, 1]	$1-\sum_i \frac{(v_i+y_i) * \frac{\min(v_i, y_i)}{\max(v_i, y_i)}}{(v_i+y_i)}$
Avg (L1, L8) Distance	2	0.01609	-433	Combination / [0, Inf)	$\frac{1}{2}\sum_i \left[\lvert y_i - v_i\rvert + \max_i\{\lvert y_i - v_i\rvert\}\right]$
J Divergence	2	0.01958	-533	Shannon's Entropy/ [0, Inf)	$\sum_i (y_{i-} - v_i)\ln\left(\frac{v_i}{y_i}\right)$
Taneja Divergence	2	0.01987	-532	Combination / [0, Inf)	$\sum_i\left[\left(\frac{y_i+v_i}{2}\right)\ln\left(\frac{y_i+v_i}{2\sqrt{y_i v_i}}\right)\right]$
Jaccard Distance	2	0.02410	-424	Inner Product / [0, 1]	$\sum_i \frac{(v_i-y_i)^2}{v_i^2+y_i^2-v_i y_i}$
Spectral Contrast Angle	2	0.02477	-419	Inner Product / [0, Inf)	$\arccos\left(\frac{\sum_i y_i v_i}{\sqrt{\sum_i {y_i}^2 \sum_i {v_i}^2}}\right)$
Chernoff Distance	2	0.02551	-460	Fidelity / (-Inf, Inf)	$\max\left(-\log\left(\sum_i (v_i^{0.1} * y_i^{0.9})^{0.9}\right)\right)$
Kullback-Leibler Divergence	2	0.02833	-590	Shannon's Entropy/ [0, Inf)	$\sum_i v_i \ln\left(\frac{v_i}{y_i}\right)$
VW6	2	0.03062	-405	Vicis Wave Hedges/ [0, Inf)	$\min\left(\sum_i \frac{(y_i-v_i)^2}{y_i}, \sum_i \frac{(y_i-v_i)^2}{v_i}\right)$
Neyman Chi Squared Distance	2	0.03077	-304	Chi Squared / [0, Inf)	$\sum_i \frac{(y_i-v_i)^2}{y_i}$
Canberra Metric	2	0.03915	-219	L1 Distance / [0, 1]	$\frac{1}{\sum_i I(v_i \neq 0)}\sum_i \frac{\lvert y_i - v_i\rvert}{(y_i+v_i)}$
Inner Product Distance	2	0.03945	-157	Inner Product / (-Inf, 1]	$1-\sum_i v_i y_i$
Euclidean Distance	2	0.04175	-33	LP Distance / [0, Inf)	$\sqrt{\sum_i \lvert y_i - v_i\rvert^2}$
Squared Euclidean Distance	2	0.04175	-346	Chi Squared / [0, Inf)	$\sum_i \lvert y_i - v_i\rvert^2$
Minkowski 3 Distance	2	0.05613	-312	LP Distance / [0, Inf)	$\sqrt[3]{\sum_i \lvert y_i - v_i\rvert^3}$
Minkowski 4 Distance	2	0.06829	-301	LP Distance / [0, Inf)	$\sqrt[4]{\sum_i \lvert y_i - v_i\rvert^4}$
Chebyshev Distance	2	0.08639	-286	LP Distance / [0, 1]	$\max_i\{\lvert y_i - v_i\rvert\}$

Gower Distance	2	0.09929	-276	L1 Distance / [0, Inf)	$\frac{1}{N}\sum_{i}\lvert y_i - v_i\rvert$
Additive Symmetric Chi Squared	3	0.09180	-53	Chi Squared / [0, Inf)	$\sum_{i}\frac{(y_i - v_i)^2(y_i + v_i)}{y_i v_i}$
VW3	3	0.09180	-53	Vicis Wave Hedges/ [0, Inf)	$\sum_{i}\frac{(y_i - v_i)^2}{\min(y_i, v_i)}$
Pearson Chi Squared Distance	3	0.09269	-52	Chi Squared / [0, Inf)	$\sum_{i}\frac{(y_i - v_i)^2}{v_i}$
VW5	3	0.09269	-52	Vicis Wave Hedges/ [0, Inf)	$\max\left(\sum_{i}\frac{(y_i - v_i)^2}{y_i}, \sum_{i}\frac{(y_i - v_i)^2}{v_i}\right)$
Kumar Johnson Divergence	3	0.11664	-28	Shannon's Entropy/ [0, Inf)	$\sum_{i}\left(\frac{({y_i}^2 - {v_i}^2)^2}{2(y_i v_i)^{3/2}}\right)$
Symmetric Chi Squared Distance	3	0.19042	-64	Chi Squared / [0, Inf)	$\sum_{i}\frac{(y_i - v_i)^2}{y_i v_i}$
VW1	3	0.19042	-64	Vicis Wave Hedges/ [0, Inf)	$\sum_{i}\frac{\lvert y_i - v_i\rvert}{\min(y_i, v_i)}$
VW2	3	0.24403	-12	Vicis Wave Hedges/ [0, Inf)	$\sum_{i}\frac{(y_i - v_i)^2}{(\min(y_i, v_i))^2}$
Whittaker Index of Association Distance	4	0.07148	-354	Shannon's Entropy/ [0, Inf)	$\frac{1}{2}\sum_{i}\left\lvert\frac{v_i}{\frac{v_i}{n}} - \frac{y_i}{\frac{y_i}{n}}\right\rvert$
Clark Distance	4	0.23491	-111	Chi Squared / [0, Inf)	$\sqrt{\sum_{i}\left(\frac{\lvert y_{i-} - v_i\rvert}{y_i + v_i}\right)^2}$
Divergence Distance	4	0.23491	-108	Chi Squared / [0, Inf)	$2\sum_{i}\frac{(y_{i-} - v_i)^2}{(y_i + v_i)^2}$
Canberra Distance	4	0.25856	-98	L1 Distance / [0, Inf)	$\sum_{i}\frac{\lvert y_i - v_i\rvert}{(y_i + v_i)}$
Wave Hedges Distance	4	0.26702	-88	Intersection / [0, Inf)	$\sum_{i}\frac{\lvert y_i - v_i\rvert}{\max\{y_i, v_i\}}$

[8] Hotea, I.; Sirbu, C.; Plotuna, A.M.; Tîrziu, E.; Badea, C.; Berbecea, A.; Dragomirescu, M.; Radulov, I. Integrating (Nutri-)Metabolomics into the One Health Tendency—The Key for Personalized Medicine Advancement. *Metabolites* **2023**, *13*, 800. https://doi.org/10.3390/metabo13070800.

[10] Koo, I.; Zhang, X.; Kim, S. Wavelet- and Fourier-transform-based spectrum similarity approaches to compound identification in gas chromatography/mass spectrometry. *Anal. Chem.* **2011**, *83*, 5631–5638. https://doi.org/10.1021/ac200740w.

Table S2. Table of the average values for each of the three most important factors in predicting cluster membership for the max scaled dataset.

Cluster	t-Statistic	Overlap Score	Score Median
1	408.187	0.028	0.240
2	-405.251	0.034	3.88×10^{11}
3	-70.745	0.134	3.33×10^{29}
4	-147.916	0.215	106.039

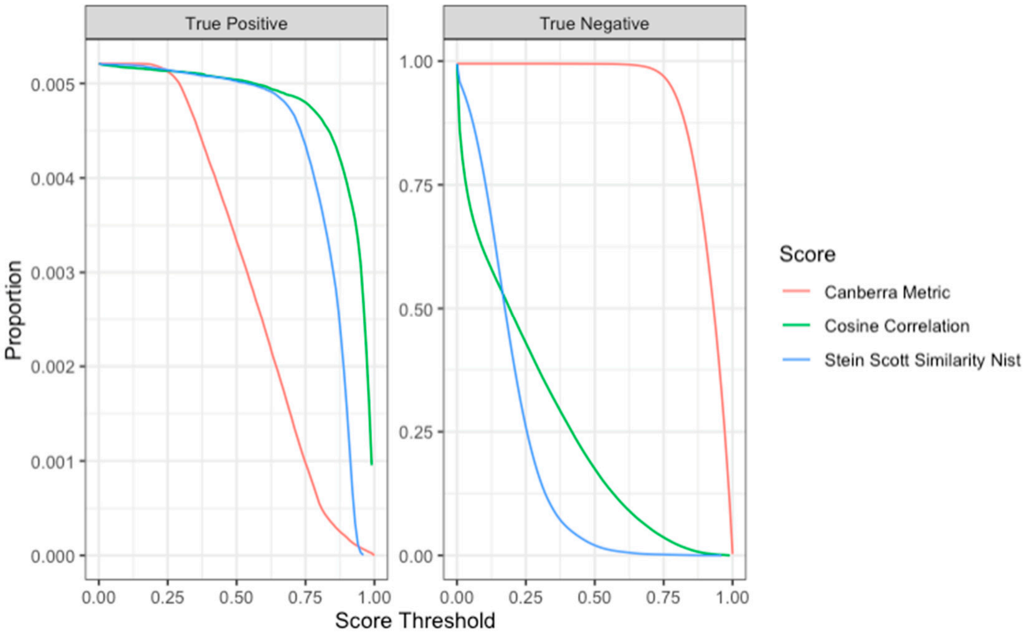

Figure S2. Proportions of true positives (left) and true negatives (right) among the combined total number of true positives and negatives above different values of score threshold. Trends in proportion are displayed for three scores: Canberra Metric (red), Cosine Correlation (green), and NIST Stein Scott Similarity (blue).
